# Evaluating the substantivity of silver diamine fluoride in a dentin model

**DOI:** 10.1002/cre2.376

**Published:** 2020-12-08

**Authors:** Brian Minavi, Adam Youssefi, Ryan Quock, Ariadne Letra, Renato Silva, Timothy C. Kirkpatrick, Gena Tribble, Ransome van der Hoeven

**Affiliations:** ^1^ Department of Endodontics University of Texas Health Science Center, School of Dentistry at Houston Houston Texas USA; ^2^ Department of Diagnostic and Biomedical Sciences University of Texas Health Science Center, School of Dentistry at Houston Houston Texas USA; ^3^ Department of Restorative Dentistry and Prosthodontics University of Texas Health Science Center, School of Dentistry at Houston Houston Texas USA; ^4^ Department of Periodontics and Dental Hygiene University of Texas Health Science Center, School of Dentistry at Houston Houston Texas USA

**Keywords:** chlorhexidine, *Enterococcus faecalis*, silver diamine fluoride, sodium hypochlorite

## Abstract

**Objectives:**

The goal of endodontic therapy is to prevent apical periodontitis. This is achieved by biomechanical preparation, microbial control using endodontic irrigants, and complete obturation of the canal space. In order to prevent possible post‐obturation complications and for an added antimicrobial effect, substantivity is a desired characteristic of endodontic irrigants. Currently the most commonly used endodontic irrigant that produces an antibacterial substantivity effect is chlorohexidine (CHX). Silver diamine fluoride (SDF) is a topically applied agent for managing dental caries and has shown to stop caries lesion progression. The objective of this study was to compare the antimicrobial substantivity effect of 3.8% SDF against other commonly used endodontic irrigants such as 2% CHX and 6.25% Sodium hypochlorite (NaOCl).

**Material and methods:**

Using a diffusion disc assay we determined the antimicrobial activities of 38%, 3.8%, 0.38%, and 0.038% of SDF against the bacterium *Enterococcus faecalis* OG1RF. Subsequently, we compared the levels of colonization of *E. faecalis* by scanning electron microscopy (SEM) at 1.5‐ and 3‐week time intervals on dentin pretreated with 3.8% SDF, 6.25% NaOCl, 2% CHX or sterile phosphate buffered saline (PBS).

**Results:**

The diffusion disc assay demonstrated that 38% and 3.8% of SDF inhibited the growth of *E. faecalis*. Moreover, the substantivity of 3.8% SDF (*p* < 0.01) was comparable to 2% CHX (*p* < 0.01) and it is significantly greater than 6.25% of NaOCl compared to the PBS treated samples after 1.5 and 3 weeks of incubation.

**Conclusions:**

In this study, we demonstrate that SDF possesses antimicrobial properties against the opportunistic pathogen *E. faecalis*. Moreover, using a dentin model we show the substantivity of 3.8% SDF is significantly greater than 6.25% NaOCl, but is comparable to 2% CHX.

## 1 INTRODUCTION

The primary goal of endodontic therapy is the preservation of the natural dentition by the elimination and or prevention of apical periodontitis. Apical periodontitis is fundamentally an inflammatory response to bacteria and their metabolic products within the root canal system (Kakehashi et al., 1965). Endodontic therapy aims to eliminate these bacteria and their products and seal the canal to prevent re‐infection. One of the steps of the process is irrigation with an antimicrobial agent. To effectively clean and disinfect the root canal system, an irrigant should be able to disinfect and penetrate dentin and its tubules, offer long‐term antibacterial effect (substantivity), remove the smear layer, and be non‐antigenic, nontoxic and non‐carcinogenic. In addition, it should have no adverse effects on dentin or the sealing ability of filling materials (Torabinejad et al., 2002). Furthermore, it should be relatively inexpensive, convenient to apply and cause no tooth discoloration (Torabinejad et al., 2002). Other desirable properties for an ideal irrigant include the ability to dissolve pulp tissue and inactivate endotoxins (Zehnder, 2006).

Sodium hypochlorite (NaOCl) is the most commonly used root canal irrigant; it is an antiseptic and inexpensive lubricant that has been used in dilutions ranging from 0.5% to 5.25% (Zehnder, 2006). Advantages of NaOCl include its ability to dissolve organic substances present in the root canal system and its affordability. The major disadvantages of NaOCl are its cytotoxicity when injected into periradicular tissues, foul smell and taste, ability to bleach clothes and to cause corrosion of metal objects (Gomes et al., 2001). In addition, it does not kill all bacteria (Shabahang & Torabinejad, 2003; Shuping et al., 2000; Siqueira Jr. et al., 1997; Sjogren et al., 1997), nor does it remove all the smear layer (McComb & Smith, 1975). It also alters the properties of dentin (Sim et al., 2001). Exposure of NaOCl to oxygen, room temperature and light can inactivate it significantly (Piskin & Turkun, 1995), making it highly reactive with the potential of the antimicrobial effect to diminish quickly over time. On the other hand, chlorohexidine (CHX) is able to persist as an antibacterial agent long after it was applied, a property called substantivity (White et al., 1997). Substantivity can aid endodontic treatment by a prolonged antimicrobial action and also to help prevent possible post‐obturation re‐infection. CHX has shown to retain substantivity for up to 12 weeks (Rosenthal et al., 2004). However, a potential drawback of CHX is the possible reactivity with NaOCl resulting in the formation and precipitation of parachloroaniline, which is cytotoxic (Basrani et al., 2010). In addition, CHX cannot dissolve organic substances and necrotic tissues present in the root canal system, it is unable to kill all bacteria and remove the smear layer (Estrela et al., 2008; Shabahang et al., 2008).

In the United States, silver diamine fluoride (SDF) has gained considerable attention in preventative and restorative dentistry (Horst & Heima, 2019). A recent systematic review and meta‐analysis revealed that yearly application of 38% SDF to exposed root surfaces of geriatric adults is a simple, inexpensive, and effective way of preventing caries initiation and progression (Oliveira et al., 2018). The only known potentially relevant side effect would be the discoloration of the treated caries lesions and dental practitioners should be prepared to address patients' concerns regarding the darkening of the decayed tooth surfaces after SDF applications (Oliveira et al., 2018). In terms of toxicity, no adverse events have been reported since the approval of the use of SDF (Chu & Lo, 2008).

Research on the use of SDF in endodontics is still lacking. Currently in Japan, an endodontic irrigant containing 3.8% SDF (Saforide 3.8%, Toyo Seiyaku Kasei Co. Ltd.) is available for root canal disinfection. The use of SDF as an endodontic irrigant is feasible as it can effectively remove microbes present in the canal and circumpulpal dentin (Mathew et al., 2012). This reagent has the potential to be used as an antimicrobial root canal irrigant or interappointment dressing, especially in locations in which potential browning or blackening of dentin by metallic silver is not a major concern (Hiraishi et al., 2010). More importantly, SDF has not shown to be cytotoxic or carcinogenic as opposed to CHX, further suggesting it to be used as a potential endodontic irrigant. However, no study has evaluated the substantivity of SDF as an endodontic irrigant compared to other commonly used endodontic irrigants.

The aims of this study were (1) to demonstrate the antimicrobial properties of SDF and (2) to test the antimicrobial substantivity effect of 3.8% SDF compared to commonly used endodontic antibacterial irrigants such as 2% CHX, 6.25% NaOCl and sterile PBS against the opportunistic pathogen *Enterococcus faecalis*.

## 2 MATERIALS AND METHODS

### 2.1 Diffusion disc assay

Overnight grown *E. faecalis* OG1RF cultures normalized to an optical density (OD) of 0.1 were spread on Brain Heart Infusion (BHI) media using a sterile applicator. Subsequently, a 10‐fold dilution series of SDF starting with 38% was prepared using sterile distilled water. The dilution series consisted of 38%, 3.8%, 0.38%, and 0.038% concentrations of SDF. Using sterile forceps, sterile paper discs (Whatman) were impregnated with each dilution of SDF and placed evenly on the spread plate. Sterilized water was used as a control. The plates were allowed to incubate for 24 hours at 37°C. Thereafter, plates were removed from the incubator and the zones of inhibition were measured using a millimeter ruler. The experiment was repeated three times.

### 2.2 Preparation of bovine dentin

Previously extracted single rooted bovine incisors with the coronal aspect of the tooth removed were obtained and maintained in sterile saline. A diamond disc was used on a slow speed motor at 30,000 RPM to remove the cementum from the root surface. Thereafter the dentin was cut into pieces with approximately 7 × 7 × 1.5 mm dimensions (length × width × thickness) using a diamond disc bur and were polished with a Kement machine and sand paper discs. All dentin specimens were placed in 6.25% NaOCl for 5 min, followed by 17% EDTA for 5 min in an ultrasonic bath, thereafter the samples were rinsed with sterile phosphate buffered saline (PBS) and autoclaved for 20 min at 121°C.

### 2.3 Dentin binding inhibition assay

Sterilized dentin specimens were place in 3.8% SDF, 6.25% NaOCl, 2% CHX or sterile PBS for 2 min. Thereafter, the samples were rinsed in Eppendorf tubes containing sterile PBS 3 times using a sterile forceps and dried on sterilized absorbent paper. The sample were subsequently placed in Eppendorf tubes containing BHI inoculated with OD = 0.1 normalized *E. faecalis*. PBS treated samples were placed in BHI in the presence of *E. faecalis* (positive control) or in the absence of the bacteria (negative control). The samples were allowed to incubate for 1.5 and 3 weeks at 37°C. Seven dentin specimens were exposed to each treatment at 1.5 and 3 weeks respectively. After 1.5 and 3 weeks of incubation, the infected and non‐infected dentin specimens were rinsed 3 times in sterile PBS and processed for scanning electron microscopy (SEM). All dentin samples for each treatment was processed.

### 2.4 Preparation of dentin samples for SEM imaging

Dentin samples were fixed in 2.5% glutaraldehyde for 30 min. Thereafter, the samples were washed in PBS 3 times and dehydrated for 10 min in ascending concentrations of ethanol (30–100% vol/vol). The samples were dehydrated in 50% ethanol: 50% t‐butanol for 10 min, followed by 100% t‐butanol for 5 min. Subsequently, the samples were allowed to dry in a desiccator for 48 hours. The dried samples were mounted on SEM stubs using conductive adhesive tape, sputter coated (Cressington 208HR) and imaged using a FEI Nova NanoSEM 230 ultra‐high‐resolution scanning electron microscope. Twenty fields of view were imaged at approximately X 50,000 magnification for each sample within the treatments. The number of bacteria were counted in each field.

### 2.5 Statistical analysis

GraphPad Prism 6.0 (GraphPad Software, San Diego California USA) was used for statistical analysis. Analysis of diffusion disc inhibition assay was perfumed using an unpaired student's *t*‐test. To analyze the dentin binding inhibition assay, analysis of variance and Student's *t*‐test was used. A *p* < 0.05 was considered statistically significant for both assays.

## 3 RESULTS

**FIGURE 1:**
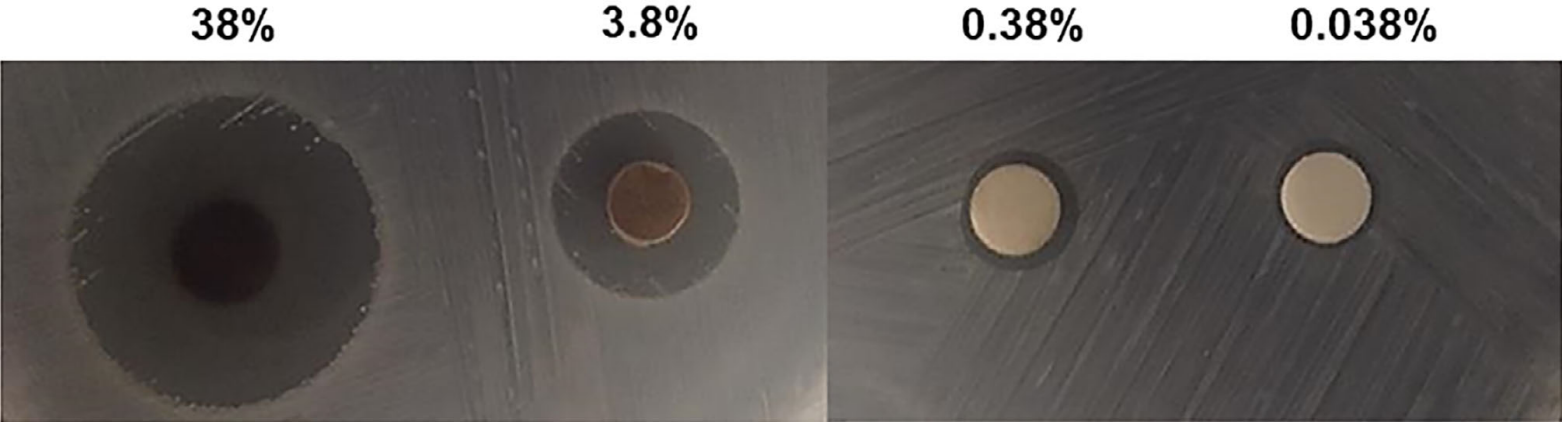
Representative images of the zones of inhibition for concentrations 38, 3.8, 0.38 and 0.038% of SDF against *Enterococcus faecalis* OG1RF. The experiment was repeated three times

**FIGURE 2:**
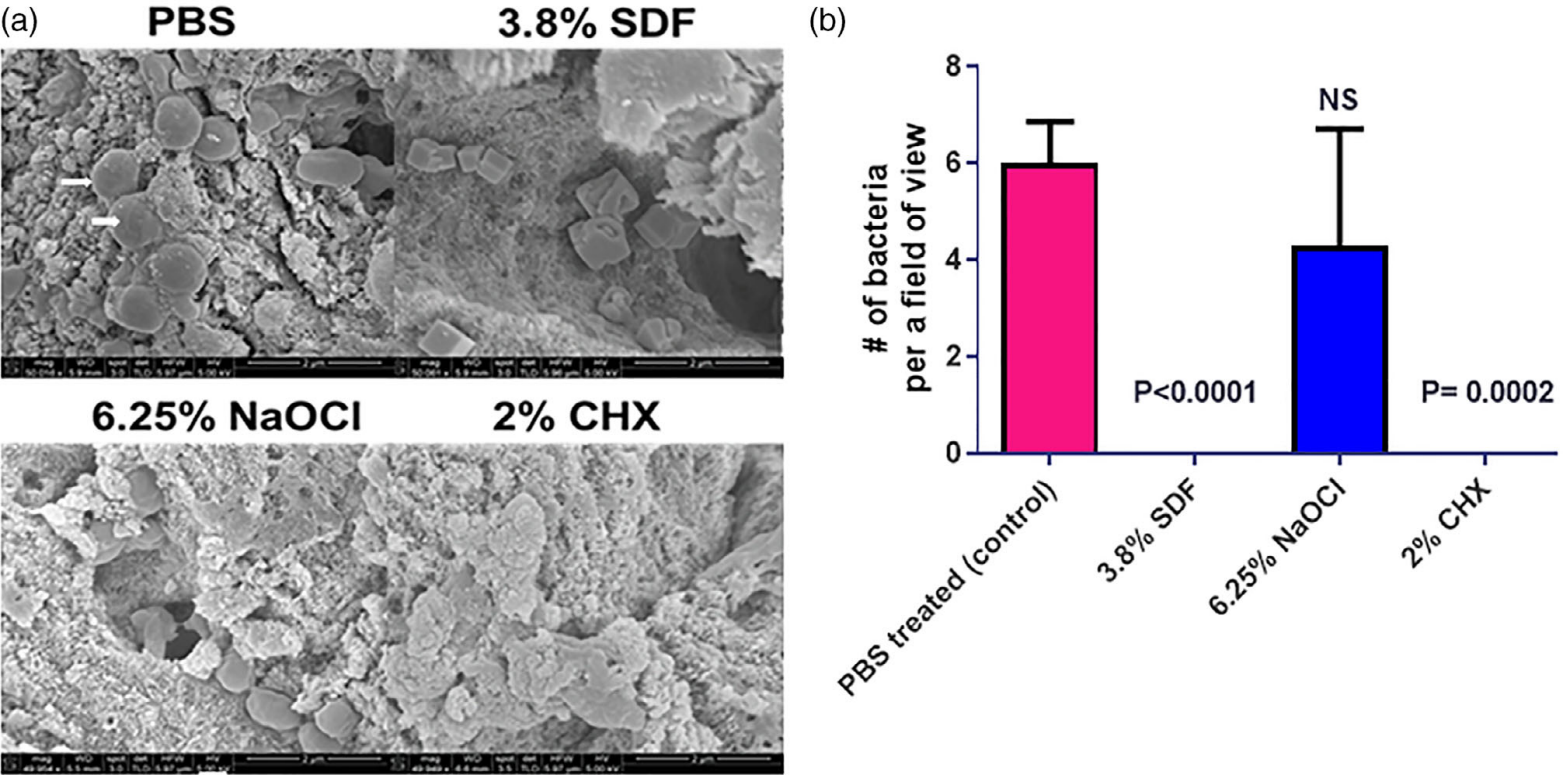
Colonization of PBS, 3.8% SDF, 6.25% NaOCl or 2% CHX pretreated dentin by *Enterococcus faecalis* OG1RF after 1.5 weeks of incubation. (a) Representative images of the colonization of *E. faecalis* on PBS, 3.8% SDF, 6.25% NaOCl or 2% CHX pretreated dentin. (b) Quantification of the number of bacteria bound to dentin in the presence of PBS, 3.8% SDF, 6.25% NaOCl or 2% CHX. A total of seven dentin samples were analyzed for each treatment (Scale bar represents 2 μm)

**FIGURE 3:**
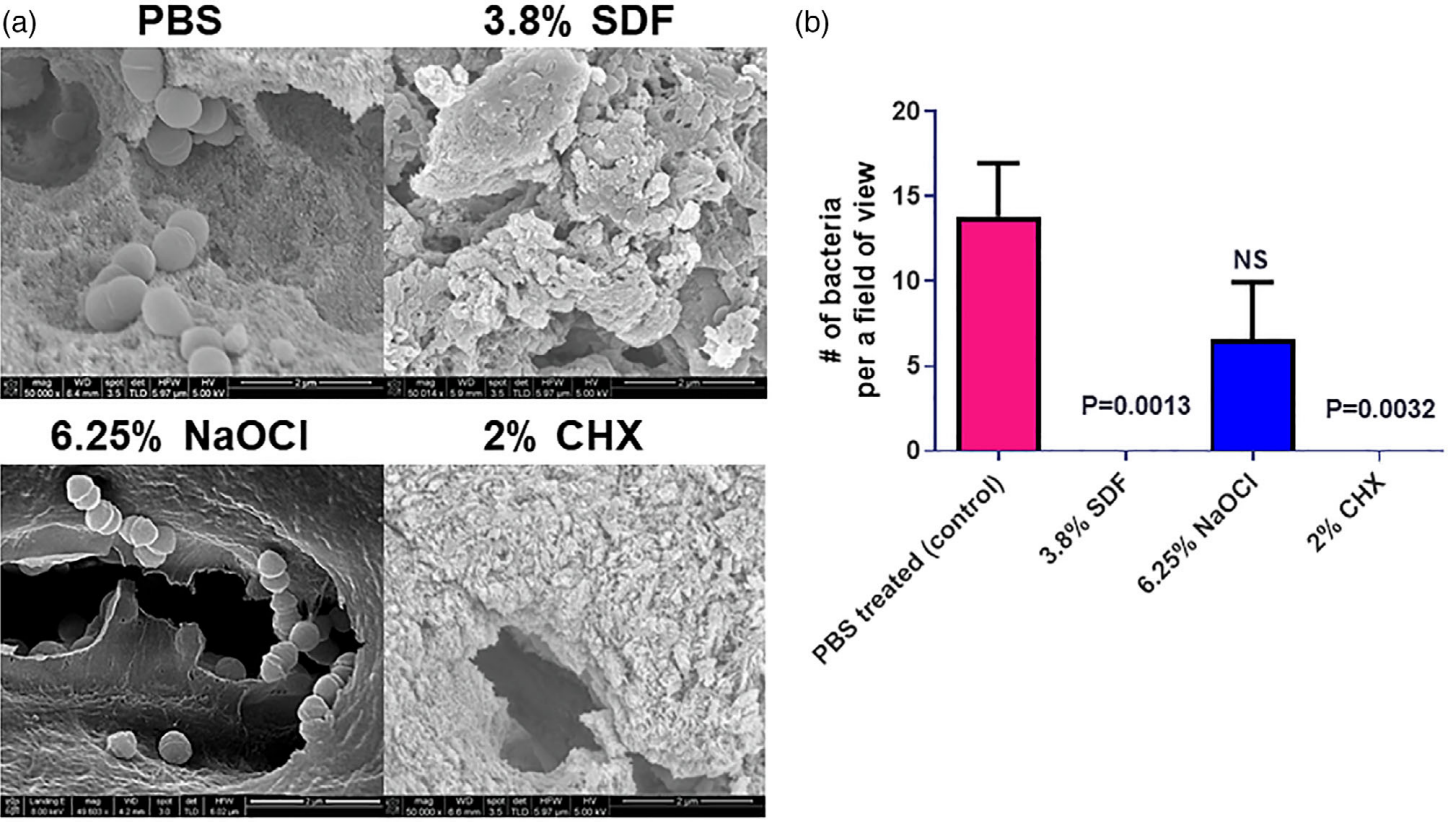
Colonization of PBS, 3.8% SDF, 6.25% NaOCl or 2% CHX pretreated dentin by *Enterococcus faecalis* OG1RF after 3 weeks of incubation. (a) Representative images of the colonization of *E. faecalis* on PBS, 3.8% SDF, 6.25% NaOCl or 2% CHX pretreated dentin. (b) Quantification of the number of bacteria bound to dentin in the presence of PBS, 3.8% SDF, 6.25% NaOCl or 2% CHX. A total of seven dentin samples were analyzed for each treatment (Scale bar represents 2 μm)

The zones of inhibition for 38%, 3.8% and 0.38% of SDF were 20 mm (±1 mm), 12 mm (±0.5 mm) and 7 mm (±1 mm), respectively (Figure 1). Our data suggests that 38% and 3.8% (*p* < 0.05) possesses antimicrobial activity against *E. faecalis*, since the zones of inhibition for 0.38% and 0.038% SDF were comparable to sterile distilled water. Using a dentin model, we observed a smaller number of bacteria bound to dentin in the 3.8% SDF treated samples compared to PBS treated samples (*p* < 0.0001) after 1.5 weeks of incubation (Figure 2a,b). Similarly, a significantly lower number of bacteria was observed on the 3.8% SDF treated samples compared to PBS treated samples (*p* = 0.0013) after 3 weeks of incubation (Figure 3a,b). The number of bacteria bound to dentin was comparable between the 3.8% SDF and 2% CHX treated samples after 1.5 and 3 weeks of incubation. However, the number of bacteria bound to the 6.25% NaOCl treated samples was similar to the PBS treated sample.

## 4 DISCUSSION

In this study we demonstrate that 38 and 3.8% SDF possesses antimicrobial properties against the opportunistic pathogen *E. faecalis*. More importantly, using a dentin model we show the substantivity of 3.8% SDF is significantly greater than 6.25% NaOCl, but is comparable to 2% CHX. These data suggest 3.8% could potentially be used an endodontic irrigant\medicament.

The need to study other irrigation solutions for endodontic treatment should be a goal of future endodontic research. This is because all endodontic solutions possess some limitations, and do not provide a solution for all endodontic treatments. Currently, the only commonly used endodontic irrigant that possesses substantivity is CHX. Substantivity can be considered as an added layer of protection against re‐infection of the root canal system via leakage of the coronal restoration (Madison & Wilcox, 1988). This will also provide a prolonged antimicrobial effect within the dentinal tubules (Rosenthal et al., 2004). However, several studies have shown that CHX reacts with NaOCl to produce a brown precipitate of parachloroaniline within the dentinal tubules. Parachloroaniline has shown to be cytotoxic and a potential carcinogen (Basrani et al., 2007; Basrani et al., 2010). Therefore, CHX is not highly recommended in all endodontic irrigation protocols. SDF has been shown to be a potential irrigant or interappointment dressing for endodontic treatment (Hiraishi et al., 2010), due to its antimicrobial properties and the absence of any cytotoxic effects. However, the question of the substantivity of SDF had not been addressed. Other studies have evaluated the anti‐microbial effect of SDF, but unfortunately most have failed to address the substantivity properly (Hiraishi et al., 2010; Mathew et al., 2012; Mei et al., 2014). Our data suggests 3.8% SDF maintains substantivity within the dentinal tubules over a period of 3 weeks, which is comparable to 2% CHX. Hence, SDF can be considered as potential irrigant/medicament to reduce the risk of reinfection in this period until a final coronal restoration is placed.

In this study, SEM of analysis of the SDF pretreated samples showed the presence of symmetric cube like structures embedded on the surface of the dentin. However, we did not observe these crystalline structures in the CHX and NaOCl pretreated dentin samples. We speculate that these structures may represent silver diamine crystals attached to the surface of the dentin. SEM in conjuncture with Energy Dispersive X‐ray Spectroscopy (EDS) could be used to further confirm the identify of these crystalline structures which may contribute to the antimicrobial and substantivity properties of this solution.

Using a dentin model, we demonstrate *E. faecalis* was unable to colonize the dentin after 1.5 and 3 weeks of incubation in the presence of 3.8% of SDF. We recognize that we are unable to ascertain if this is due to the bacteriostatic or bactericidal properties of SDF or due to the displacement of the bacteria by the disposition of the crystalline material on the surface of dentin. Another limitation of the study is that we only used *E. faecalis* OG1RF to test the substantivity of SDF. The use of other endodontic bacteria can further substantiate the antimicrobial and substantivity properties of this potential irrigant/medicament. We also recognize the duration of the study may be a limitation to clearly establish the substantivity of SDF. The maintenance of viable in vitro biofilms for longer durations of time is challenging and may not provide a solution to ascertain the true substantivity of SDF. The use of a rodent model may provide an opportunity to assess the substantivity of SDF. Nevertheless, using the dentin model we established that 3.8% SDF has comparable substantivity to 2% CHX and may provide an alternative to CHX.

## 5 CONCLUSION

Our study demonstrates that 3.8% SDF possesses antimicrobial properties and substantivity comparable to 2% CHX. However, further studies need to be completed to demonstrate if SDF can be used in combination with other commonly used endodontic irrigants, such as NaOCl.

## AUTHOR CONTRIBUTIONS

Brian Minavi, Ryan Quock, Ariadne Letra, Renato Silva, Timothy Kirkpatrick, Gena Tribble and Ransome van der Hoeven contributed to design of the study, while Brian Minavi, Adam Youssefi and Ransome van der Hoeven were involved in the collection, analysis and interpretation of the data. Brian Minavi and Ransome van der Hoeven wrote the original draft of the manuscript. Ryan Quock, Ariadne Letra, Renato Silva, Timothy Kirkpatrick and Gena Tribble critically revised the manuscript and provided important intellectual content. All authors approved the final version of the manuscript.

## ACKNOWLEGMENTS

We thank Dr. Danielle Garsin (University of Texas, Health Science Center at Houston, McGovern Medical School) for providing the *E. faecalis* OG1RF strain. We also like to thank Dr. Jianhua (James) Gu (Electron Microscopy Core, Houston Methodist) for assisting with the SEM imaging and Dr. Gilberto Garcia for helping with the extraction and preparation of the bovine teeth. This work was supported by startup funds provided by the University of Texas, Health Science Center, School of Dentistry at Houston to R. van der Hoeven.

## CONFLICT OF INTEREST

The authors declare that they have no conflict of interest.

## Data Availability

Data sharing not applicable to this article as no datasets were generated or analysed during the current study.
